# Biological Safety: Principles and Practices, 5th Edition

**DOI:** 10.3201/eid2501.180458

**Published:** 2019-01

**Authors:** Imke Schröder

**Affiliations:** University of California at Los Angeles, Los Angeles, California, USA

**Keywords:** biosafety, best practices, pathogens, recombinant DNA

The 5th edition of *Biological Safety: Principles and Practices* (Figure) is still the leading comprehensive biosafety textbook available and is a page-turner as well. The book extensively covers the identification, assessment, and management of biological hazards, as well as special environments as they relate to biohazardous substances. It broadly deals with bacterial, viral, and fungal pathogens; biological toxins; and recombinant DNA used in academic research, medical, pharmaceutical, and veterinary laboratories. The book provides extensive background on the biohazards and details the risk to humans, animals, and, if applicable, to plants. For example, in the chapter “Viral Agents of Human Disease: Biosafety Concerns,” Rozo, Lawler, and Parags present an overview of the viral life cycle, epidemiology, and diversity. They also summarize the clinical manifestation of viral disease and provide extensive examples of viral classes known for laboratory-associated infections, with information on postaccident management.

**Figure:**
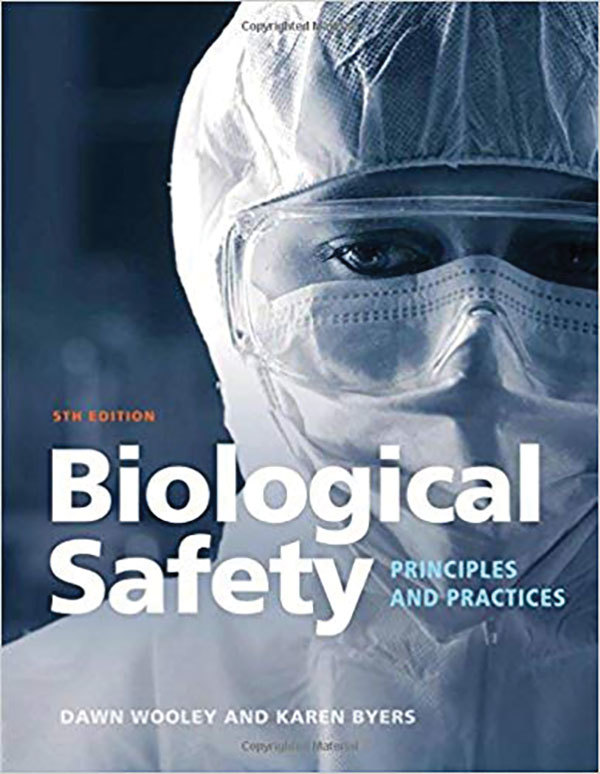
Biological Safety: Principles and Practices

The 5th edition has added 8 new chapters covering topics that gained relevance in more recent years, such as a chapter on molecular agents describing genome editing, the use of recombinant T-cells for cancer therapy, and prions. Other new chapters deal with specialized biocontainment for research on virus-transmitting mosquitoes and research on aerosolized microorganisms. The 5th edition also expands on training programs, veterinary and greenhouse biosafety, field studies, and clinical laboratory safety.

*Biological Safety: Principles and Practices* stands out as a safety textbook because it is not purely focused on regulatory requirements, and it is enjoyable to read. All chapters are supplemented with useful tables summarizing essential information, and many contain descriptive diagrams. I appreciated the Laboratory Animal Allergy Questionnaire and the sample Biosafety Level 2 checklists, which can be very useful for faculty who begin to work in these areas. Furthermore, the chapters provide numerous references to relevant current publications and regulatory guidelines from the National Institutes of Health and Centers for Disease Control and Prevention. There is some overlap in chapters regarding details on bacterial virulence factors, viruses, and biosafety cabinets, which could be consolidated should a revision be considered.

The attention to detail and the assemblage of leading experts contributing to this book is clearly a labor of love by editors Dawn Wooley and Karen Byers. Both additionally authored chapters; for example, Dawn Wooley, who is well known for her work on risk assessment of viral vectors, contributed to chapters on molecular agents and on risk assessment of biologic hazards. Karen Byers lent her expertise to a chapter on laboratory-acquired infections, an area she has researched extensively. Because the material extends beyond academic research and includes clinical and veterinary laboratory practices, it is also appropriate for medical and veterinary personnel. Finally, the book can easily serve as a textbook for biosafety courses and even complement microbiology courses. 

I found all topics of the book highly engaging and worth reading; the concisely written chapters can be read alone according to area of interest or serve as a reference for questions that might arise. The book is evidence based and illustrates data-driven best practices. It provides a modern view of biosafety practices; as such, it is a valuable resource not only for biosafety professionals but also for researchers working with biohazards.

